# Have We an Infallible Treatment for Diphtheria?

**Published:** 1890-02

**Authors:** F. E. Waxham

**Affiliations:** Chicago, Illinois; Professor of Rhinology and Laryngology in the College of Physicians and Surgeons, Chicago


### HAVE WE AN INFALLIBLE TREATMENT FOR DIPHTHERIA?*

By F. E. WAXHAM, M. D., CHICAGO, ILLINOIS.

Professor of Rhinology and Laryngology in the College of Physicians and Surgeons, Chicago.

From time to time certain remedies have been brought before the attention of the profession and the public, and the most exorbitant claims made as to their efficacy. Many of these remedies have been found useful, some useless, some positively inju-

*Read before the Chicago Medical Society, January 6, 1890.

rious, and none at all entitled to the name of *specific*. Recently a line of treatment has been proposed and advocated with the greatest earnestness, zeal and eloquence.* It not only has been advocated as infallible, but the declaration even made that the ordinary preventive measures were unnecessary. Such teaching should not pass unchallenged, and I therefore crave your indulgence and raise my voice in opposition.

There is no specific remedy for diphtheria, and there never can be one, and this can honestly be said without any discredit to the medical profession.

All accidents and physical injuries vary in degree from the most trifling to the most appalling.

All attacks of diphtheria in like manner vary in intensity; some mild and insignificant and unassociated with danger, others again of the most malignant and virulent type and entirely beyond our control. Not only this, but each individual varies, in resisting power, in inherent vitality. A disease that kills one patient may be recovered from in another, under the same identical treatment, on account of this greater vitality. It is just as unreasonable, just as absurd to believe that we can cure every case of diphtheria as it is to believe that we can prevent death in every case of accident. True it is that in case of physical injury we can often prevent a fatal issue. We can ligate the severed artery, we can set the broken limb, we can dress the wound antiseptically and often conduct a case to safe recovery that otherwise would surely perish. The same is true of diphtheria; by careful nursing, by the *early* and *thorough* application of the most approved methods of treatment and by timely surgical interference we can save many a case that would just as surely perish; but to claim that all cases can be cured and that we have an infallible remedy, is asserting more than human power can accomplish. In proof of these assertions I would report two cases coming under my observation where death occurred in spite of the most rigid employment of so-called infallible treatment.

CASE I.—I was called December 20th, 1889, in consultation by Dr. Geo. E. Willard to see a boy between two and three years

*Dr. A. E. Hoadley, in *Times and Register*, December 2, 1889.

of age, and learned that the patient had been sick three days with diphtheria. He had a year and a half previously suffered from a very severe attack of diphtheria and croup from which he recovered after being desperately sick. He had recently been in good health, and his present attack seemed only of moderate severity. Upon examination it was observed that the uvula was elongated and with some membrane formation upon it; some exudation was also present upon the lateral walls of the pharynx and the nasal cavities somewhat involved. The pulse was good, the child bright and altogether the case seemed a hopeful one; so much so that a favorable prognosis was given. The patient had been from the first upon full and frequent doses of iron and general supporting treatment. In view of the fact that an infallible remedy had been proposed we felt it our duty to give it a trial, and it was remarked by Dr. Willard that this would certainly be a fair and safe case in which to test it. In place of other treatment the child was given the following mixture:

℞. Tr. myrrhæ,........................................ʒiii.

Potass. chl............................................ ʒi.

Acid. carbolici,......................................gr. iv.

Mel. despumat,......................................ʒiv.

Aquae q. s. ad,... .....................................℥iv. M.

Sig.: One-half teaspoonful every half hour. The same mixture was used as a spray for the throat and diluted one-half and also used as a wash for the nasal cavities. Stimulation and proper nourishment were also insisted upon. The medicine was given with religious fidelity, but from the time of its administration the patient began to fail; still the medicine was continued faithfully and regularly, but in spite of its infallibility the patient died within twenty-four hours.

CASE II.—December 21, 1889, I was called by Dr. E. R. Bennett to operate upon a patient with diphtheritic croup, but upon arrival found the child dead. Dr. Bennett has kindly given me the following history:

"Ida R., aged 2 years and 2 months, had always been considered delicate by the parents. Had pharyngeal diphtheria six weeks before and recovered. Was called to attend her last illness December 19, 1889. History given was a slight fever, very marked restlessness, a distinctly hoarse, ringing cough with no expectoration. These symptoms only existed twenty-four hours before my arrival. The difficulty of breathing increased rapidly. Inspection of fauces showed a diphtheritic deposit covering both tonsils. Diagnosis, diphtheritic croup. Prognosis, grave.

Commenced immediately the administration of the myrrh mixture as advocated. Also administered the juice of a fresh pineapple, a teaspoonful every half hour. Inhalations of the vapor of lime were repeated at intervals, and the atmosphere of the apartment was moist constantly with the same. In spite of all medication the voice rapidly became extinct, the pulse failed, and patient expired on the 21st, three days after the onset of the disease."

In this case operative measures were postponed on account of the faith in the infallibility of the treatment. I have since learned that subsequently another child in the same family was taken with the disease and was in an alarming condition, but have not been informed as to the result.

It may be said that no conclusions can be drawn from the report of two cases Two fatal cases, however, are sufficient to destroy the reputation of any line of treatment for infallibility.

In conclusion I would again repeat that we have no specific for diphtheria, and would insist upon still clinging to old and well established methods of treatment. The *frequent* use of iron in full doses, free stimulation, abundance of nourishment, watchful care, antiseptic gargles and washes for the throat and nose, strychnia and digitalis in case of depression, and the bichloride of mercury when the larynx becomes invaded,—these remedies are our sheet anchors in the treatment of diphtheria, and no specific remedy can displace them; while isolation, ventilation, and disinfection are safeguards that should *never*, *never*, be omitted.

